# GC/MS Analysis and Protective Effects of *Mentha longifolia* L. Essential Oil Against Antituberculosis Drug-Induced Organs Toxicity in Wistar Albino Rats

**DOI:** 10.3390/plants13223231

**Published:** 2024-11-17

**Authors:** Usama K. Abdel-Hameed, Abdulaziz S. Abualghaith, Shaza H. Aly, Mohamed Mostafa Soliman, Lamiaa Adnan Munshi, Safia A. A. Mohammed, Omayma A. Eldahshan, Eman A. R. Abdelghffar

**Affiliations:** 1 Biology Department, College of Science, Taibah University, Al-Madinah Al-Munawara 42353, Saudi Arabia; usama_abdelhameed@sci.asu.edu.eg (U.K.A.-H.); aabualghaith@taibahu.edu.sa (A.S.A.); lmunshi@taibahu.edu.sa (L.A.M.); saamohammed@taibahu.edu.sa (S.A.A.M.); 2 Botany Department, Faculty of Science, Ain Shams University, Cairo 11566, Egypt; 3 Department of Pharmacognosy, Faculty of Pharmacy, Badr University in Cairo (BUC), Cairo 11829, Egypt; shaza.husseiny@buc.edu.eg; 4 Department of Biology, College of Science, Jazan University, P.O. Box. 114, Jazan 45142, Saudi Arabia; msoliman@jazanu.edu.sa; 5 Zoology Department, Faculty of Science, Ain Shams University, Cairo 11566, Egypt; 6 Pharmacognosy Department, Faculty of Pharmacy, Ain Shams University, Cairo 11566, Egypt; 7 Center for Drug Discovery Research and Development, Ain Shams University, Cairo 11566, Egypt

**Keywords:** antituberculosis, cytokines, GC/MS, hepatotoxicity, *Mentha longifolia*, silymarin

## Abstract

*Mentha longifolia* (L.) L., also known as wild mint, is a perennial herbaceous plant that belongs to the Lamiaceae family. This study aimed to investigate the effects of essential oil of *M. longifolia* (MLEO) on oxidative stress and inflammatory responses in the liver and kidneys in the context of drug-induced liver injury caused by the anti-TB drugs rifampicin, isoniazid, and pyrazinamide (INH-RIF-PZA). The chemical composition of MLEO was characterized using GC/MS analysis, which revealed the presence of pulegone, *trans*-*p*-menthan-3-one, piperitenone, and *β*-caryophyllene as its major volatile constituents. An INH/RIF/PZA mixture was administered to Wistar rats for 30 days, and silymarin was administered as a standard drug. MLEO was administered p.o. at doses of 50 mg and 100 mg/kg b.w. Both doses of the MLEO therapy effectively regulated all biochemical indicators of hepatic impairment and reduced the damage caused by the INH/RIF/PZA mixture. It may be deduced that MLEO has the ability to protect organs against INH/RIF/PZA-induced damage and could potentially be a valuable natural remedy for treating anti-TB-induced liver and kidney injuries.

## 1. Introduction

Tuberculosis (TB) is a highly prevalent disease that impacts approximately one-third of the global population [1]. The causative agent of tuberculosis is *Mycobacterium tuberculosis*, a pathogen that leads to a severe and often fatal infection, causing widespread illness and mortality [2]. Approximately 10 million individuals received a TB diagnosis in 2020. The 30 nations with the highest prevalences accounted for 86% of the estimated cases of tuberculosis worldwide [3].

Tuberculosis is classified into latent tuberculosis and active tuberculosis. Latent tuberculosis refers to a tuberculosis infection where the person does not exhibit any symptoms because the *Mycobacterium* is inactive [4]. On the other hand, active tuberculosis is contagious, as it manifests symptoms and can readily spread to others. Tuberculosis is a prominent cause of mortality worldwide. Further, nearly a quarter of the global population is affected by tuberculosis [5].

The World Health Organization (WHO) has recognized multidrug-resistant tuberculosis (MDR-TB) as a significant risk to worldwide health, with prevalence rates of around 3.4% among those with new cases of TB and 18% among those who have been treated before. Countries with underdeveloped health systems and scarce resources, particularly in sub-Saharan Africa, Eastern Europe, and Asia, endure the greatest burden of MDR-TB [6].

Isoniazid (INH) and rifampicin (RIF) are the primary medications used in the initial treatment of tuberculosis (TB). Nevertheless, the potential liver damage induced by these pharmaceuticals is a significant worry during clinical treatment, particularly due to the extended length of therapy and the simultaneous use of multiple medications [7]. RIF, as a powerful stimulator of CYP2E1, has the potential to worsen INH-induced liver toxicity by enhancing the generation of harmful metabolites like hydrazine. This leads to a synergistic effect, increasing the liver damage caused by INH [8]. Another drug utilized is Pyrazinamide (PZA), which, upon metabolism, transforms into pyrazinoic acid (PA), resulting in the development of granulomatous hepatitis [9]. Therefore, a mixture of three main anti-TB drugs (RIF/INH/PZA) causes up to 30% of hepatotoxicity that ranges from liver function test abnormalities to acute liver failure [10]. Drug resistance, the exorbitant cost of tuberculosis medications, and unpleasant effects pose significant challenges in the treatment of tuberculosis [11].

The adverse effects linked to anti-TB drugs prompted drug researchers to shift their focus toward natural alternatives [12,13,14,15]. Medicinal plants have garnered significant global interest due to their diverse biological activities and potential applications in various diseases [16,17,18,19]. This has led the scientific community to explore their potential as novel drug candidates for preventing and treating the ranges of toxicity associated with diseases, including tuberculosis [20,21,22].

*Mentha longifolia* (L.) L. (wild mint) is an herbaceous perennial plant belonging to the family Lamiaceae. The term “habek” is well recognized for *M. longifolia* in the Jazan area [23]. It is indigenous to the continent of Europe, western and central Asia, and northern and southern regions of Africa, excluding tropical areas [24]. This plant is utilized to address common ailments like coughs, colds, stomach discomfort, asthma, indigestion, and headaches. Additionally, it displays effectiveness in healing injuries and reducing swollen glands [23]. Moreover, many pharmacological studies have demonstrated therapeutic benefits of *M. longifolia* in various areas, including antioxidant, antibacterial, antifungal, anti-inflammatory, hepatoprotective, and anti-diarrheal properties [25,26,27]. Phytochemical investigations of *M. longifolia* revealed it is mainly rich in volatile oil, in addition to flavonoids and phenolic acids [24,28,29].

Milk thistle (*Silybum marianum*) is the source of silymarin, a flavonoid compound that has long been known to have strong hepatoprotective properties. Studies on both humans and animals have clearly shown how effective it is at shielding the liver from a variety of harms, including those caused by chemicals, medications, and illnesses. Silymarin’s well-established reputation makes it a valuable reference drug for assessing the hepatoprotective and renoprotective properties of other herbal remedies that show promise for future research and potential therapeutic applications [30,31]. There is some evidence that peppermint essential oil (PEO) may effectively prevent drug-induced hepatorenal injuries through techniques such as scavenging reactive oxygen species (ROS). Active components of PEO, such as menthol and menthone, are powerful antioxidants that contribute to the scavenging of ROS and prevent subsequent damage to kidney and liver cells due to oxidative stress. Consuming PEO also inhibits proinflammatory cytokines, which might be associated with activating the Nrf2 pathway, an anti-inflammatory pathway [32,33,34,35,36].

This study was designed to explore the effect of *M. longifolia* essential oil (MLEO) on toxicity to different organs, including the liver and kidneys, and their oxidative stress and inflammatory response statuses during anti-TB drug-induced organ toxicity.

## 2. Results

### 2.1. GC/MS Analysis of Essential Oil of M. longifolia

**Figure 1:**
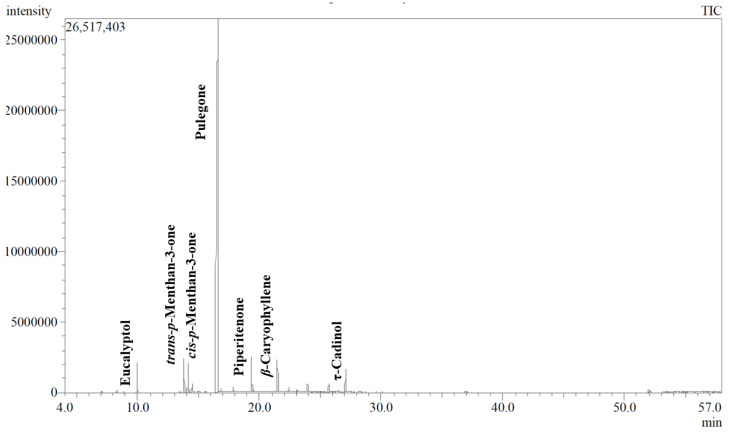
GC chromatogram of essential oil isolated from aerial parts of *M. longifolia*.

Essential oil isolated from *M. longifolia* was analyzed using GC/MS to identify its chemical composition, which is detailed in Table 1. The GC/MS analysis of essential oil of *M. longifolia* identified eighteen compounds, representing about 99.86% of the total oil. The major identified compounds were found to be pulegone (82.02%) along with trans-p-menthan-3-one (2.92%), piperitenone (2.69%), and β-caryophyllene (2.19%). The oil predominantly comprised oxygenated monoterpenes (93.39%) along with sesquiterpene hydrocarbons (3.40%) and oxygenated sesquiterpenes (2.77%). A GC chromatogram of the essential oil is presented in Figure 1.

### 2.2. In Vivo Effects of M. longifolia Essential Oil Against Anti-TB Drug-Induced Organ Toxicity

#### 2.2.1. The Effects of *M. longifolia* Essential Oil on Body and Organ Weight

The current findings show that, in comparison to the normal control group, the induction of hepatotoxicity by the anti-TB drug mixture (INH/RIF/PZA) decreased body weight (*p* < 0.001) but increased (*p* < 0.001) the weights of the liver and kidneys as well as their relative weights (Table 2). Concurrent oral administration of either SILY or two doses of MLEO significantly increased body weight (*p* < 0.05–0.001) and decreased the weights of the liver and kidneys as well as their relative weights (*p* < 0.001) when compared with the anti-TB drug mixture group. Moreover, there were no significant differences between the SILY group and the high-dose MLEO group, while there were statistically significant increases in the weights of the liver and kidneys as well as their relative weights in the SILY group compared to the low-dose MLEO group (*p* < 0.05–0.001).

#### 2.2.2. The Effects of *M. longifolia* Essential Oil on Biochemical Markers

Administration of an anti-TB drug mixture (INH/RIF/PZA) produced marked impairment of liver and kidney function, as shown by significant increases (*p* < 0.001) in serum ALP, ALT, AST, LDH, GGT, total cholesterol, triglycerides, LDL-Chol., vLDL-Chol., urea, uric acid, and creatinine levels as well as cardiac risk indices I and II (*p* < 0.001) in comparison to the normal control group (Table 3). In addition, serum HDL-Chol. and the total antioxidant capacity were significantly increased (*p* < 0.001) in the anti-TB drug mixture (INH/RIF/PZA) group in comparison to the control group.

Concurrent oral administration of either SILY or two doses of MLEO significantly decreased (*p* < 0.001) the elevated levels of serum ALP, ALT, AST, LDH, GGT, total cholesterol, triglycerides, LDL-Chol., vLDL-Chol., urea, uric acid, and creatinine as well as cardiac risk indices I and II when compared with the anti-TB drug mixture (INH/RIF/PZA) group. Similarly, administration of either SILY or two doses of MLEO significantly increased (*p* < 0.001) the serum level of HDL-Chol. and the total antioxidant capacity when compared with the anti-TB drug mixture (INH/RIF/PZA) group. Moreover, there were no significant differences between the SILY group and the high-dose MLEO group, while there were statistically significant differences between the SILY group and the low-dose MLEO group (*p* < 0.05–0.001) in serum GGT, lipid profile, kidney function, and total antioxidant capacity.

#### 2.2.3. The Effects of *M. longifolia* Essential Oil on Oxidant/Antioxidant Markers

The levels of hepatorenal antioxidants such as SOD, GSH-PX, CAT, and GSH were significantly decreased (*p* < 0.001). In contrast, hepatorenal MDA and NO levels were significantly increased (*p* < 0.001) in the anti-TB drug mixture (INH/RIF/PZA) group in comparison to the normal control group (Table 4). Concurrent oral administration of either SILY or two doses of MLEO significantly increased (*p* < 0.001) the hepatorenal SOD, GSH-PX, CAT, and GSH levels. Still, it decreased (*p* < 0.001) the levels of hepatorenal MDA and NO when compared with the anti-TB drug mixture (INH/RIF/PZA) group. Moreover, there were no significant differences between the SILY group and the high-dose MLEO group, in contrast, there were statistically significant differences between the SILY group and the low-dose MLEO group (*p* < 0.05–0.001) in serum MDA and NO, as well as hepatorenal SOD, GSH-PX, CAT, and GSH levels.

#### 2.2.4. The Effects of *M. longifolia* Essential Oil on Serum Inflammatory Markers

The levels of serum inflammatory markers such as TNF-α, IL-1β, IL-6, and NF-κB were significantly increased (*p* < 0.001) in the anti-TB drug mixture (INH/RIF/PZA) group, while the IL-10 and adiponectin levels were significantly decreased (*p* < 0.001) in the anti-TB drug mixture (INH/RIF/PZA) group in comparison to the normal control group (Table 5). Concurrent oral administration of either SILY or two doses of MLEO significantly decreased (*p* < 0.001) the TNF-α, IL-1β, IL-6, and NF-κB levels and significantly increased (*p* < 0.001) the IL-10 and adiponectin levels compared with the anti-TB drug mixture (INH/RIF/PZA) group. Moreover, there were no significant differences between the SILY group and the high-dose MLEO group. At the same time, there were statistically significant differences between the SILY group and the low-dose MLEO group (*p* < 0.05–0.001) in serum TNF-α, IL-1β, IL-6, NF-κB, IL-10 and adiponectin levels.

## 3. Discussion

Hepatic and renal impairment are major concerns throughout tuberculosis treatment. The liver breaks down antituberculosis medications into hazardous, reactive metabolites, including hydrazine. These metabolites then combine to form ROS, stimulating lipid peroxidation; disrupting the antioxidant defense mechanism; causing inflammation in cells; and finally causing loss of membrane integrity, intracellular component leakage, and cell death. Because the kidneys are closely related to the liver as excretory organs, antituberculosis medications not only induce abnormalities in the liver, which is the primary site of detoxification, but also negatively impact the kidneys. Kidney damage occurs because they are exposed to the hazardous effects of intermediate or completed toxic metabolites [1,2,8,10]. Consequently, the primary goal of the current study was to investigate efficient treatments to repair the hepatorenal impairment caused by antituberculosis drugs. Functional foods can either prevent disease symptoms from occurring or reduce their severity. Globally, there is increased interest in therapies with natural sources like foods and plants with medicinal properties.

In agreement with our results, previous research on an MLEO widely cultivated in Madinah revealed that the dominant constituents were pulegone (38.42%), eucalyptol (15.60%), menthone (13.20%), and isopulegone (9.81%) [37]. Another study on oil of *M. longifolia* subsp. cyprica collected in Northern Cyprus revealed the presence of pulegone (64.8%) as a major identified compound [38].

A further investigation indicated that *M. longifolia* exhibited significant concentrations of cineole and pulegone, comprising 33.4% and 40.7%, respectively [39]. These findings varied from those of essential oil of *M. longifolia* L. subsp. schimperi cultivated in Sudan, which exhibited high concentrations of carvone and limonene, comprising 67.3% and 13.5%, respectively [40]. In another study, oil extracted from *M*. *longifolia* cultivated in Tunisia had significant concentrations of pulegone (26.92%), 1.8 cineole (21.3%), and L-menthone (10.66%) during the winter season. In the spring, the oil displayed major components including pulegone (38.2%) and two fatty acids, oleic and palmitic acids, which comprised 23.79% and 15.26%, respectively [41]. A separate investigation of *M. longifolia* collected from Albania indicated that the oil exhibited a comparable composition characterized by cineole, linalool, menthol, pulegon, carvone, piperthone, and *β*-caryophyllene [42].

We can conclude that the principal constituents found in *M. longifolia* essential oil are pulegone, 1,8-cineole, menthone, menthol, carvone, limonene, piperitone, and piperitenone oxide with different concentrations based on the geographical source, environmental variations, and agronomical factors [37,43,44].

Pulegone and piperitenone are monoterpene ketones found in several mint species that may have antioxidant, anti-inflammatory, and cytoprotective properties [43,45]. Pulegone, the main constituent of MLEO, exerts a strong inhibitory effect on the synthesis of nitric oxide (NO), as well as the expression of inducible nitric oxide synthase (iNOS) and cyclooxygenase-2 (COX-2). Furthermore, a Western blot analysis demonstrated that pulegone decreased the activation of NF-κB and MAPKs induced by lipopolysaccharide (LPS) in RAW 264.7 cells [45]. Moreover, another study showed that pulegone has antioxidant properties and hepatoprotective efficacy during CCl_4_-induced toxicity [46].

Pulegone decreased cholesterol, triacylglycerides, and lipid oxidation. In addition, it also raised the SOD, CAT, GSH, GPx, and antioxidant vitamin levels, and it was able to counteract the harmful effects of CCl_4_ in rats [46]. In LPS-treated RAW 264.7 cells, pulegone efficiently scavenged ROS and suppressed NF-κB in a dose-dependent manner to prevent the formation of NO and proinflammatory genes such as TNF-α, IL-1β, and IL-6, without causing any cytotoxic effects in the cells [45]. A previous study reported that peppermint oil caused significant reductions in the elevated liver enzymes ALT and AST. Significant decreases were also seen in lipid peroxidation parameters in rats with non-alcoholic fatty liver disease [47]. Other previous findings revealed that a peppermint oil rat group had dramatically reduced lipid peroxidation (MDA), ALT, and AST and significantly increased final body weights, body weight gain, GSH, and GPX compared to CCl_4_ groups. In addition, peppermint oil was found to drastically reduce uric acid and creatinine levels. Stabilization of the plasma membrane and healing of tissue damage were shown by decreases in the serum levels of AST, ALT, and ALP following therapy with peppermint oils [48].

Another prior study found that pretreatment with silymarin (as a reference drug) or peppermint leaf essential oil before administering CCl_4_ significantly decreased stress parameters such as ALT, AST, ALP, urea, creatinine, total cholesterol, triglycerides, and LDL and increased the HDL level in comparison to a group that received CCl_4_ alone. In addition, rats treated with peppermint leaf essential oil showed considerable decreases in hepatic and kidney lipid peroxidation (TBARS) and increases in the antioxidant enzymes SOD, CAT, and GPx in comparison to rats treated with CCl_4_ [32]. The presence of sesquiterpenes such as caryophyllene and caryophyllene oxide, which are known to have excellent anti-inflammatory properties, may contribute to the anti-inflammatory activity of peppermint essential oil [32].

Another prior investigation found that administering *M. longifolia* essential oils to rats may restore all hepatic and plasma indicators to normal levels in animal models of sepsis, including lipid peroxidation, ALT, AST, and GSH. These findings suggested that essential oils of *M. longifolia* could effectively treat liver damage [49].

The menthol and menthone in peppermint essential oil can enhance the antioxidant defense system, control inflammatory processes, and suppress liver injuries [50]. A report suggests that the hepatoprotective effect may be attributed to flavonoids and steroids [11]. The significant hepatoprotective benefits seen in this study may be attributed to flavonoids found in *Mentha longifolia*. Furthermore, peppermint essential oil can greatly reduce the risk of kidney damage caused by gentamicin without compromising this drug’s antibacterial properties [33]. The hepatoprotective, antioxidant, anti-inflammatory, and antitoxic properties of Mentha species make them valuable medicinal agents [51]. Phenolic chemicals are among the bioactive molecules found in the genus Mentha. Many of these substances have gained notoriety due to their antioxidant properties, which enhance the removal of ROS [33,52].

Hepato- and nephrotoxicity are also associated with systemic inflammation. The protective action of adiponectin against liver damage is partially attributed to its antagonistic impact on TNF-α. Adiponectin has several anti-inflammatory effects, such as lowering TNF-α and direct effects on inflammatory cells as well as NF-κB, one of the key modulators of expression of proinflammatory genes like TNF-α, IL-1β, and IL-6 [53,54]. Additionally, adiponectin induces the anti-inflammatory cytokine-like IL-10, contributing to its anti-inflammatory properties [54]. The hepatoprotective activity of MLEO is implied by its action, which is like that of SILY in that it decreases proinflammatory markers and elevates adiponectin. As observed in several previous studies, Mentha’s antioxidant capacity and anti-inflammatory qualities may account for some of its beneficial effects [52,55]. *M. longifolia* is widely distributed in nature, representing a wonderful fountain of inspiration, as it is full of different plants exhibiting multiple biological activities and potential medicinal uses [30,56,57].

In several experimental models, the essential oil of *Mentha* sp., which contains many bioactive components such as monoterpenes and sesquiterpenes, has shown encouraging antioxidant, anti-inflammatory, hepatoprotective, and renoprotective qualities [47,52,54,55]. Research has demonstrated the potency of this essential oil in notably mitigating oxidative stress, inflammation, lipid peroxidation, and cellular injury in the liver and kidneys—important processes implicated in the toxicity of antituberculosis drugs. Examining natural substances like *M. longifolia* essential oil as possible supplemental therapies might be a worthwhile research endeavor, especially considering the rising concern about the negative effects of anti-TB medications, particularly on the liver and kidneys. More clinical trials are necessary to confirm their safety and effectiveness when treating human TB.

## 4. Materials and Methods

### 4.1. Plant Material

The aerial parts of *M. longifolia* (Lamiaceae) were collected from Al-Madinah Al-Munawarah, Kingdom of Saudi Arabia, in October 2021. Usama K Abdelhameed kindly identified and authenticated the plant. Voucher plant specimen with code UKAKSA01 was kept at the Department of Pharmacognosy, Ain Shams University, Cairo, Egypt, and the Department of Biology, College of Science, Taibah University, KSA.

### 4.2. Isolation of Essential Oil of Mentha longifolia

The fresh aerial parts of *M. longifolia* (100 g each) were finely cut and hydrodistilled for 5 h using a Clevenger apparatus. After hydrodistillation, the essential oil was isolated and kept in a sealed glass tube at −4 °C until GC/MS analysis [58]. The obtained oil was colorless with a pleasant aroma, and the average yield was 0.2% (*v*/*w*).

### 4.3. Gas Chromatography–Mass Spectrometry Analysis (GC/MS)

A Shimadzu GCMS-QP 2010 chromatograph (Kyoto, Japan) equipped with a DB-5 capillary column (30 m × 0.25 mm i.d. × 0.25 μm film thickness; Restek, Centre County, PA, USA) was used to perform gas chromatography/mass spectrometry (GC/MS) analysis. The injector temperature was 250 °C, while the oven temperature was 45 °C for 2 min, then changed to 30 °C at 5 °C/min and maintained at 300 °C for 5 min. Helium was the carrier gas and flowed at 1.40 mL/min. To inject diluted samples (1% *v*/*v*), a 15:1 split ratio was used, and the volume was 1 μL. The operational parameters of the MS were as follows: the temperature of the interface was 280 °C, the temperature of the ion source was 220 °C, the electron ionization (EI) mode was set to 70 electron volts (eV), and the scan range was from 35 to 500 atomic mass units (amu). The volatile constituents were identified by correlating their retention indices, comparing their fragmentation patterns with the NIST Mass Spectral Library and the Wiley Library database, and referencing published data in the literature [59,60,61,62,63,64]. The Kovats retention indices (RIs) were determined by comparing them to a series of n-alkanes (C8–C30) that were injected using identical conditions.

### 4.4. Compound Identification

Identification was achieved by comparing the Kovats retention index, along with mass spectrometric data (molecular ion peaks and fragmentation patterns), to the information recorded in the NIST Mass Spectral Library under similar conditions [15,65,66,67].

### 4.5. Animals and Experimental Design

**Figure 2:**
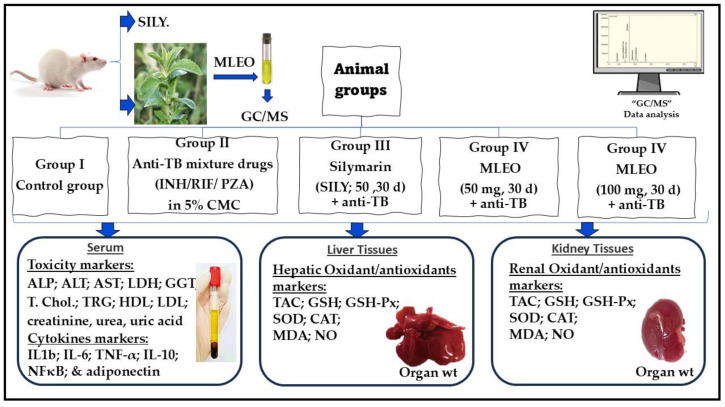
Experimental design.

Adult male Wistar albino rats from the College of Pharmacy weighing 120 ± 5 g were maintained under normal laboratory conditions. They were kept in standard polypropylene cages at a room temperature of 25 ± 5 °C and provided a standard diet and water ad libitum. The College of Pharmacy of Taibah University, al Madina, KSA, approved this study (COPTU-REC-102-20240927). Five groups, each with six rats, were used, and they were treated as follows (Figure 2):

Group I served as the negative control group and received the vehicle (5% carboxymethylcellulose (CMC) in an isotonic saline solution (ISS) at a volume not exceeding 10 mL/kg) orally daily for 30 days.

Group II served as the positive control group and received an anti-TB drug mixture (INH/RIF/PZA) to induce injuries in organs like the liver and kidneys. The anti-TB drug mixture [rifampicin (RIF, 54 mg/kg; from Novartis Pharma Company, Amreya, Egypt); isoniazid, also known as isonicotinic acid hydrazide (INH (27 mg/kg; from El Nasr Company for Chemicals and Drugs, Cairo, Egypt)); and pyrazinamide (PZA, 135 mg/kg; from Amoun Pharmaceutical Co., Cairo, Egypt)] was solubilized in 5% CMC in an ISS and administered orally daily for 30 days [67].

Group III received silymarin (SILY; 50 mg/kg, p.o., for 30 days; from Novartis Pharma, Cairo, Egypt) concurrent with an anti-TB drug mixture and served as the reference group [31].

Group IV received *M. longifolia* essential oil (MLEO) (50 mg/kg b.w., p.o., for 30 days) [48] concurrent with an anti-TB drug mixture.

Group V received *M. longifolia* essential oil (MLEO) (100 mg/kg b.w., p.o., for 30 days) [48] concurrent with an anti-TB drug mixture. The doses for the antitubercular medicines were extrapolated from daily human doses using a conversion table [67,68].

Finally, at the end of the experiment, the animals were anesthetized (ketamine/xylazine), and blood samples were collected by heart puncture from all animals and centrifuged at 3000 rpm for 10 min to obtain plasma. Liver and kidney samples were immediately transferred to ice-cold containers and homogenized in an appropriate buffer using a homogenizer. The homogenates were used to measure the biochemical parameters. In addition, initial body weight, final body weight, and weight gain were measured.

### 4.6. Chemicals and Kits

#### 4.6.1. Liver/Kidney Function and Lipid Profile

Serum alkaline phosphatase (ALP; CAT No. MBS841841), alanine transaminase (ALT; CAT No. MBS169579), aspartate transaminase (AST; CAT No. MBS9719085), lactate dehydrogenase (LDH; CAT No. MBS822351), and gamma-glutamyltransferase (GGT; CAT No. MBS9719035) were analyzed by available colorimetric assay kits (MyBioSource, Inc., San Diego, CA, USA). Serum total cholesterol (T. Chol.; CAT No. 024-500), triglycerides (TRG; CAT No. 059L-100), high-density lipoprotein cholesterol (HDL-Chol.; CAT No. 041-050), and low-density lipoprotein cholesterol (LDL-Chol.; CAT No. 047-050) were measured using commercial kits from UDI (United Diagnostic Industry, Dammam, K.S.A.). Very low-density lipoprotein cholesterol (VLDL-Chol) was calculated according to a previous study [68] as VLDL = {Triglycerides ÷ 5}. Serum creatinine (CR; CAT No. CR3814), urea (UR, CAT No. UR3825), and uric acid (UC, CAT No. UA3824) were estimated using diagnostic kits (Randox Laboratories, Crumlin, UK) following the manufacturer’s instructions.

#### 4.6.2. Oxidant/Antioxidant Markers

Total antioxidant capacity (TAC; CAT No. MBS2556554), reduced glutathione (GSH; CAT No. MBS9718981), glutathione peroxidase (GSH-Px; CAT No. MBS9718985), superoxide dismutase (SOD; CAT No. MBS8819950), catalase (CAT; CAT No. MBS2556989), malondialdehyde (MDA; CAT No. MBS2540409), and nitric oxide (NO; CAT No. MBS480450) were measured by commercially available colorimetric kits (MyBioSource, Inc., San Diego, CA, USA) following the manufacturer’s instructions.

The cytokine markers serum interleukin-1 beta (IL1b; CAT No. MBS2023030), interleukin-6 (IL-6; CAT No. MBS451945), tumor necrosis factor-alpha (TNF-α; CAT No. MBS508536), interleukin-10 (IL-10; CAT No. MBS2503826), nuclear factor-kappaB (NFκB; CAT No. MBS287521), and adiponectin (Adipo.; CAT No. MBS8244709) were measured according to the manufacturer’s instructions using ELISA kits (MyBioSource, Inc., San Diego, CA, USA).

### 4.7. Statistical Analysis

The given data were all represented as means ± SDs. ANOVA, or one-way analysis of variance, was employed to examine the statistical significance among the groups. Tukey’s test for multiple comparisons was performed using Prism 6 software (Graph Pad, San Diego, CA, USA). *p* values < 0.05 were regarded as significant.

## 5. Conclusions

The current study shows that, because of their unique chemical components, MLEOs have many methods by which their antioxidant qualities might mitigate oxidative stress and ameliorate systemic inflammation, including scavenging free radicals, modulating antioxidant enzymes, and reducing lipid peroxidation. These results support the possible advantages of employing MLEOs as adjuvant therapeutic agents in the treatment of hepatorenal toxicity and associated disorders. Treating organ toxicity through MLEOs appears promising because of recent strategies used in the development of these natural and efficacious agents for improving the health of people suffering from this chronic disease, although more experiments are needed.

## Figures and Tables

**Table 1:** Chemical composition (%) of essential oil isolated from *M. longifolia* leaves using GC/MS analysis.

No.	Retention Time (Rt Min)	Compound	Retention Index		Molecular Formula	Peak Area (%)
			Exp.	Rep.		
1	9.98	Eucalyptol	1031	1031	C_10_H_18_O	1.98
2	13.81	***trans*-*p*-Menthan-3-one**	1156	1155	C_10_H_18_O	**2.92**
3	14.13	*cis*-*p*-Menthan-3-one	1166	1164	C_10_H_18_O	1.87
4	14.23	Isoborneol	1170	1172	C_10_H_18_O	0.31
5	14.48	isopulegone	1178	1175	C_10_H_16_O	0.80
6	16.58	**Pulegone**	1250	1257	C_10_H_16_O	**82.02**
7	16.87	Piperitone	1260	1260	C_10_H_16_O	0.35
8	17.86	Bornyl acetate	1296	1296	C_12_H_20_O_2_	0.45
9	19.35	**Piperitenone**	1348	1347	C_10_H_14_O	**2.69**
10	21.48	***β*-Caryophyllene**	1426	1429	C_15_H_24_	**2.19**
11	22.38	*α*-Caryophyllene	1461	1462	C_15_H_24_	0.33
12	23.10	Germacrene D	1489	1485	C_15_H_24_	0.14
13	23.95	*γ*-Muurolene	1523	1524	C_15_H_24_	0.66
14	24.17	*cis*-Calamenene	1532	1537	C_15_H_22_	0.08
15	25.71	Caryophyllene oxide	1592	1592	C_15_H_24_O	0.81
16	26.47	Epicubenol	1624	1624	C_15_H_26_O	0.15
17	27.08	τ-Cadinol	1651	1651	C_15_H_26_O	1.81
18	51.98	Untriacontane	3086	3100	C_31_H_64_	0.30
Oxygenated Monoterpenes (%)						93.39
Sesquiterpene Hydrocarbons (%)						3.40
Oxygenated Sesquiterpenes (%)						2.77
Aliphatic Hydrocarbons (%)						0.30
Total identified compounds (%)						**99.86**

The compounds are listed according to their elution in the DB-5 GC column. Major compounds are in bold. Identification was based on comparisons of the compounds’ mass spectral data (MS) and retention indices (RIs) with those of the NIST Mass Spectral Library (2011), the Wiley Registry of Mass Spectral Data (8th edition), and the literature.

**Table 2:** Effect of MLEO on body and organ weights and serum biochemical parameters during antitubercular drug-induced organ toxicity in rats.

	Groups	Control	Anti-TB Only	SILY + Anti-TB	MLEO_50_ + Anti-TB	MLEO_100_ + Anti-TB
Parameters						
Initial body weight (g)		122.5 ± 0.95	123.7 ± 0.93	126.2 ± 1.11	123.4 ± 0.90	125.4 ± 1.67
Final body weight (g)		254.4 ± 1.58	233.7 ± 1.84 ***	244.7 ± 1.78 ** ###	239.2 ± 1.28 ***	243.3 ± 1.56 *** ##
Body weight gain (g)		131.9 ± 1.53	110.0 ± 1.63 ***	118.5 ± 1.06 *** ##	115.9 ± 0.82 *** #	117.9 ± 1.44 *** ##
Liver weight (g)		5.29 ± 0.10	7.87 ± 0.06 ***	5.80 ± 0.08 * ###	6.63 ± 0.14 *** ### $$$	5.87 ± 0.18 * ###
Relative liver weight (g/100 g b.w)		2.08 ± 0.04	3.37 ± 0.04 ***	2.36 ± 0.04 ** ###	2.77 ± 0.07 *** ### $$$	2.41 ± 0.07 ** ###
Kidney weight (g)		0.59 ± 0.007	0.84 ± 0.004 ***	0.63 ± 0.007 ** ###	0.71 ± 0.005 *** ### $$$	0.62 ± 0.005 * ###
Relative kidney weight (g/100 g b.w)		0.23 ± 0.003	0.36 ± 0.004 ***	0.25 ± 0.004 *** ###	0.30 ± 0.003 *** ### $$$	0.25 ± 0.003 ** ###

Anti-TB: antitubercular medicines, MLEO: *M. longifolia* essential oil * *p* < 0.05 vs. Control group, ** *p* < 0.01 vs. Control group, & *** *p* < 0.001 vs. Control group. # *p* < 0.05 vs. Anti-TB only group, ## *p* < 0.01 vs. Anti-TB only group, & ### *p* < 0.001 vs. Anti-TB only group. $$$ *p* < 0.001 vs. SILY + Anti-TB group.

**Table 3:** Effect of MLEO on serum liver function, lipid profile, kidney function, and total antioxidant capacity during antitubercular drug-induced organ toxicity in rats.

	Groups	Control	Anti-TB Only	SILY + Anti-TB	MLEO_50_ + Anti-TB	MLEO_100_ + Anti-TB
Parameters						
ALP (IU/L)		112.1 ± 2.9	269.3 ± 2.1 ***	124.0 ± 1.4 ** ###	131.5 ± 1.2 *** ###	123.8 ± 1.4 ** ###
ALT (IU/L)		44.8 ± 1.0	92.9 ± 1.2 ***	52.0 ± 1.7 * ###	58.2 ± 2.9 *** ###	52.3 ± 1.2 * ###
AST (IU/L)		28.0 ± 1.1	83.0 ± 1.9 ***	36.8 ± 1.8 * ###	42.6 ± 2.7 *** ###	36.4 ± 1.7 * ###
LDH (μmol/L)		230.9 ± 8.4	620.8 ± 11.0 ***	281.4 ± 11.3 * ###	309.0 ± 13.5 *** ###	277.5 ± 10.4 * ###
GGT (IU/L)		12.83 ± 0.75	66.14 ± 1.67 ***	17.20 ± 0.71 * ###	33.24 ± 0.89 *** ### $$$	18.01 ± 0.66 * ###
Total cholesterol (mg/dL)		191.7 ± 2.2	320.1 ± 3.3 ***	209.3 ± 3.4 ** ###	251.0 ± 4.1 *** ### $$$	209.5 ± 3.4 ** ###
Triglycerides (mg/dL)		73.1 ± 1.7	122.5 ± 1.4 ***	83.3 ± 2.1 ** ###	98.4 ± 2.4 *** ### $$$	84.3 ± 1.7 ** ###
HDL-Chol. (mg/dL)		51.3 ± 2.1	16.1 ± 0.6 ***	43.0 ± 1.6 ** ###	28.8 ± 1.6 *** ### $$$	43.8 ± 1.5 * ###
LDL-Chol. (mg/dL)		125.8 ± 1.2	279.4 ± 3.4 ***	149.7 ± 4.2 *** ###	202.5 ± 4.8 *** ### $$$	148.8 ± 3.6 ** ###
vLDL-Chol. (mg/dL)		14.6 ± 0.3	24.5 ± 0.3 ***	16.7 ± 0.4 ** ###	19.7 ± 0.5 *** ### $$$	16.9 ± 0.3 ** ###
Cardiac Risk Index-I (CRI-I)		3.8 ± 1.2	20.0 ± 0.7 ***	4.9 ± 0.2 ###	8.9 ± 0.6 *** ### $$$	4.8 ± 0.2 ###
Cardiac Risk Index-II (CRI-II)		2.5 ± 0.1	17.4 ± 0.7 ***	3.5 ± 0.2 ###	7.2 ± 0.5 *** ### $$$	3.4 ± 0.2 ###
Urea (mg/dL)		19.5 ± 0.4	46.6 ± 2.2 ***	26.7 ± 0.4 ** ###	31.7 ± 1.0 *** ### $	27.0 ± 0.7 ** ###
Uric acid (mg/dL)		6.0 ± 0.02	15.1 ± 0.07 ***	7.1 ± 0.22 ** ###	9.8 ± 0.19 *** ### $$$	7.1 ± 0.28 ** ###
Creatinine (mg/dL)		0.9 ± 0.10	4.6 ± 0.13 ***	1.5 ± 0.10 ** ###	3.4 ± 0.11 *** ### $$$	1.4 ± 0.03 ** ###
Total antioxidant capacity (nmol/L)		1.1 ± 0.04	0.4 ± 0.02 ***	0.9 ± 0.02 ** ###	0.7 ± 0.03 *** ### $$$	1.0 ± 0.03 * ###

Anti-TB: antitubercular medicines, MLEO: *M. longifolia* essential oil, ALP: alkaline phosphatase, ALT: alanine transaminase, AST: aspartate transaminase, LDH: lactate dehydrogenase, HDL-chol.: high-density lipoprotein cholesterol, LDL-chol.: low-density lipoprotein cholesterol, VLDL-chol.: very low-density lipoprotein cholesterol. * *p* < 0.05 vs. Control group, ** *p* < 0.01 vs. Control group,& *** *p* < 0.001 vs. Control group. ### *p* < 0.001 vs. Anti-TB only group. $ *p* < 0.05 vs. SILY + Anti-TB group, & $$$ *p* < 0.001 vs. SILY + Anti-TB group.

**Table 4:** Effect of MLEO on oxidant/antioxidant parameters in liver and kidneys during antitubercular drug-induced organ toxicity in rats.

	Groups	Control	Anti-TB Only	SILY + Anti-TB	MLEO_50_ + Anti-TB	MLEO_100_ + Anti-TB
Parameters						
Liver tissues						
Nitric oxide (nmol/mg prot.)		7.55 ± 0.18	15.06 ± 0.64 ***	9.30 ± 0.35 * ###	11.54 ± 0.21 *** ### $	9.28 ± 0.32 * ###
TBARS (nmol MDA/mg prot.)		2.01 ± 0.07	5.97 ± 0.21 ***	2.85 ± 0.17 * ###	4.16 ± 0.21 *** ### $$$	2.90 ± 0.19 * ###
GSH (µmol/mg prot.)		12.68 ± 0.16	6.73 ± 0.17 ***	11.10 ± 0.36 ** ###	9.04 ± 0.22 *** ### $$$	11.27 ± 0.38 * ###
SOD (U/mg prot.)		17.92 ± 0.38	2.27 ± 0.26 ***	15.64 ± 0.41 ** ###	7.66 ± 0.49 *** ### $$$	16.00 ± 0.37 * ###
GPX (U/mg prot.)		4.61 ± 0.31	1.05 ± 0.13 ***	3.30 ± 0.23 ** ###	2.28 ± 0.14 *** ## $	3.54 ± 0.20 * ###
CAT (U/mg prot.)		176.50 ± 3.85	90.15 ± 2.39 ***	156.60 ± 3.32 ** ###	123.90 ± 1.54 *** ### $$$	161.70 ± 3.75 * ###
Kidney tissues						
Nitric oxide (nmol/mg prot.)		16.83 ± 0.26	29.23 ± 0.46 ***	18.58 ± 0.47 * ###	20.64 ± 0.45 *** ## $	18.61 ± 0.37 * ###
TBARS (nmol MDA/mg prot.)		6.26 ± 0.17	19.77 ± 0.33 ***	8.08 ± 0.39 ** ###	12.57 ± 0.37 *** ### $$$	8.17 ± 0.31 ** ###
GSH (µmol/mg prot.)		8.51 ± 0.20	4.72 ± 0.16 ***	7.45 ± 0.19 ** ###	6.27 ± 0.14 *** ### $$$	7.61 ± 0.19 * ###
SOD (U/mg prot.)		23.15 ± 0.45	7.50 ± 0.23 ***	20.92 ± 0.54 ** ###	11.74 ± 0.16 *** ### $$$	21.28 ± 0.47 * ###
GPX (U/mg prot.)		9.88 ± 0.31	5.48 ± 0.13 ***	8.36 ± 0.29 ** ###	6.96 ± 0.17 *** ## $$	8.71 ± 0.27 * ###
CAT (U/mg prot.)		83.85 ± 2.07	25.15 ± 1.4 ***	66.60 ± 3.32 ** ###	52.20 ± 3.27 *** ## $	71.73 ± 3.75 * ###

Anti-TB: antitubercular medicines, MLEO: *M. longifolia* essential oil. * *p* < 0.05 vs. Control group, ** *p* < 0.01 vs. Control group, & *** *p* < 0.001 vs. Control group. ## *p* < 0.01 vs. Anti-TB only group, & ### *p* < 0.001 vs. Anti-TB only group. $ *p* < 0.05 vs. SILY + Anti-TB group, $$ *p* < 0.01 vs. SILY + Anti-TB group, & $$$ *p* < 0.001 vs. SILY + Anti-TB group.

**Table 5:** Effect of MLEO on serum TNF-α, IL-1β, IL-6, IL-10, NF-κB, and adiponectin levels during antitubercular drug-induced organ toxicity in rats.

	Groups	Control	Anti-TB Only	SILY + Anti-TB	MLEO_50_ + Anti-TB	MLEO_100_ + Anti-TB
Parameters						
Serum TNF-α (pg/L)		14.98 ± 0.10	30.29 ± 0.30 ***	16.33 ± 0.22 * ###	20.52 ± 0.37 *** ### $$$	16.29 ± 0.25 * ###
Serum IL-1β (pg/L)		9.61 ± 0.17	22.25 ± 0.56 ***	11.64 ± 0.24 ** ###	15.95 ± 0.26 *** ### $$$	11.37 ± 0.45 * ###
Serum IL-6 (pg/L)		18.14 ± 0.43	55.53 ± 0.86 ***	21.48 ± 0.80 * ###	30.30 ± 0.58 *** ### $$$	20.93 ± 0.49 * ###
Serum IL-10 (pg/L)		34.35 ± 0.58	11.43 ± 0.33 ***	30.94 ± 0.81 * ###	22.23 ± 0.38 *** ### $$$	31.87 ± 0.71 * ###
Serum NF-κB (pg/L)		0.54 ± 0.02	1.61 ± 0.06 ***	0.77 ± 0.05 * ###	1.13 ± 0.06 *** ### $$$	0.78 ± 0.05 * ###
Serum adiponectin (pg/L)		130.30 ± 1.11	81.82 ± 1.57 ***	123.00 ± 1.22 ** ###	100.60 ± 1.01 *** ### $$$	124.40 ± 1.34 * ###

Anti-TB: antitubercular medicines, MLEO: *M. longifolia* essential oil. * *p* < 0.05 vs. Control group, ** *p* < 0.01 vs. Control group, & *** *p* < 0.001 vs. Control group. ### *p* < 0.001 vs. Anti-TB only group. $$$ *p* < 0.001 vs. SILY + Anti-TB group.

## Data Availability

Data are available upon request.
